# Prolonged Trapping of Adeno-Associated Virus Capsids Reveals that Genome Packaging Affects Single-Ion Mass Spectrometry Measurements

**DOI:** 10.1021/jacs.4c13393

**Published:** 2025-03-24

**Authors:** Eduard H. T. M. Ebberink, Victor C. Yin, Evolène Deslignière, Arjan Barendregt, Tobias P. Wörner, Kyle L. Fort, Alexander A. Makarov, Albert J. R. Heck

**Affiliations:** † Biomolecular Mass Spectrometry and Proteomics, Bijvoet Center for Biomolecular Research and Utrecht Institute for Pharmaceutical Sciences, University of Utrecht, Padualaan 8, 3584 CH Utrecht, The Netherlands; ‡ Netherlands Proteomics Center, Padualaan 8, 3584 CH Utrecht, The Netherlands; § Thermo Fisher Scientific GmbH, Hanna-Kunath-Street 11, 28199 Bremen, Germany

## Abstract

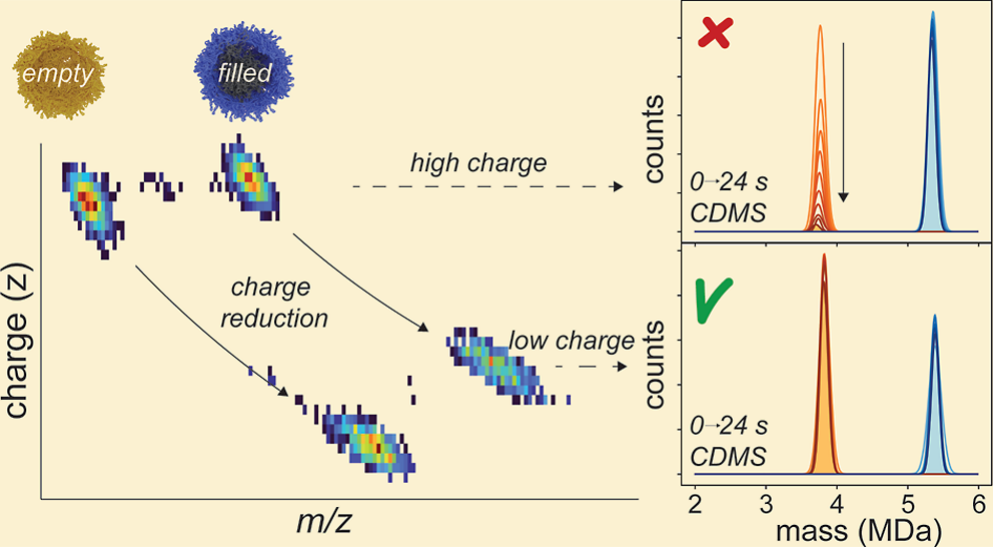

Recombinant adeno-associated viruses (rAAVs) play an important role in gene therapy, yet the optimal preparation of these biotherapeutics remains challenging, with often incomplete incorporation of genome cargo, negatively affecting therapeutic use. Genome packaging has traditionally been difficult to investigate. Charge detection mass spectrometry (CDMS) has gained prominence, as it provides the ability to mass analyze and resolve empty and filled rAAVs, enabling the quantitative determination of empty-to-filled rAAV ratios. Such measurements require a high mass resolving power and depend on the unbiased detectability of the distinct rAAV particles. The mass resolving power in the Orbitrap mass analyzers scales with transient recording times. Therefore, we extended the capability of recording ions from 1 or 2 to 24 s. When we record these 24 s transients to analyze rAAVs, we not only observe a substantial improvement in accuracy and mass resolution but also find that this can lead to erroneous artifacts in the empty-to-filled ratios. Artifacts originate from the distinct behavior between orbiting ions from either empty or filled particles, mainly due to differences in desolvation and charge losses. Ions from empty rAAVs appear much more susceptible to charge losses, which negatively affect their tracing, artificially decreasing the empty-to-filled ratios. Further elucidating the causes of this undesirable ion behavior, we provide the means to minimize charge losses, thus regaining accurate AAV quantification at an increased charge precision and mass-resolving power. As the Orbitrap-based CDMS has become a method of choice to determine this important quality control attribute, our findings are important to avoid reporting incorrect empty-to-filled ratios.

## Introduction

Recombinant adeno-associated viruses (rAAVs) are extensively used for gene therapy due to their favorable properties (e.g., nonpathogenicity, low immunogenicity, broad tropism, and long-term expression).^1−3^ The preparation of therapeutic rAAVs is hampered by the unwanted coproduction of empty capsids that do not contain the single-stranded DNA (ssDNA) gene of interest. Such empty rAAV capsids can elicit an immune response, while they are not useful.^4,5^ Therefore, quality control of the cargo load of rAAVs is important to ensure clinical-grade preparations. For therapeutic applications, the assessment of the genomic content of rAAVs is a critical attribute, which is, however, quite challenging to measure accurately. To address this issue, others and we recently explored single-particle-based methods like mass photometry (MP) and charge-detection mass spectrometry (CDMS).^6−11^ Using these methods, various populations of particles with distinct genetic filling could be distinguished and quantitatively assessed. Assessing different serotypes, different genomic cargoes, and batches of AAVs, empty, partially filled, and overfilled capsids were recurrently observed next to the desired single transgene-containing (filled) rAAV particles.^6,7,9,12^ With Orbitrap-based CDMS recently made commercially available, the method rapidly gains popularity, both in academia and industry, especially in the area of analyzing critical quality attributes (CQAs) of rAAVs, such as the genetic filling.^7,13−22^

Here, we explore the validity and accuracy of such measurements, analyzing in detail the gas-phase behavior of ions in the Orbitrap mass analyzer, especially when trapping them for extended times with the aim of boosting sensitivity and mass resolving power. When analyzing high-mass, high-inertia particles like rAAVs in the gas phase, such as with native MS or CDMS, the ions need to be transmitted to the detector, which requires semiharsh conditions during ionization, collisional cooling for efficient transmission, ion trapping, and high energy activation to desolvate and guide the ions appropriately to the mass analyzer. All of these parameters can strongly affect the particle detectability in the mass analyzer. Desolvation of particles or charge loss events can lead to loss of ion signal and broadening of detected peaks, a phenomenon described for a variety of Fourier transform (FT)-based mass spectrometry approaches (e.g., FT-ICR,^23^ Orbitrap-based CDMS,^7,24,25^ and linear ion traps^26−28^). We hypothesized that such phenomena in native (Orbitrap-based) CDMS might induce a bias in the quantitative determination of empty-to-filled ratios in rAAV particles. Therefore, we now set out to investigate how empty and genome-loaded filled capsids are potentially affected by in vacuo treatment during CDMS.

Commonly, in biomolecular mass spectrometry, peptides and proteins are trapped in the Orbitrap analyzer for just tens to thousands of milliseconds to record a transient signal. While ions of peptides and small molecules typically decay quite dramatically over this period, macromolecular particles (e.g., >1 MDa protein assemblies, AAV capsids) appear to be substantially more stable and can be contained for up to several seconds within an Orbitrap analyzer.^24^ Such extended trapping times allow longer transients to be recorded, which theoretically should lead to improved signal-to-noise and mass-resolving power.^24^ Therefore, we modified a Thermo Scientific Q Exactive UHMR mass spectrometer and combined it with an external data processing system (the FTMS Booster X2) to perform ultralong transient, enhanced single-ion CDMS.^24,29^ In a proof-of-concept, we demonstrated that about 20 times longer transients (up to 24 s) could be recorded, resulting in significant improvements in signal-to-noise and charge and single-ion mass resolving power.

The improved setup also allows an in-depth analysis of the stability of ions in the Orbitrap analyzer. As a result, we set out to analyze the potentially different ion behavior of empty and filled rAAV particles under conditions of extended ion trapping in Orbitrap-based CDMS. Remarkably, ions from empty and filled rAAVs were found to behave rather differently during the longer trapping times, negatively affecting the proper quantification of empty-to-filled ratios. Following a comprehensive study of the rAAV ion behavior in the Orbitrap analyzer, we propose a way to accommodate and optimally make use of ultralong transient CDMS for the improved analysis of rAAV masses and empty-to-filled ratios.

## Results

### Mass Resolving Power Improves with Prolonged Trapping Times but Affects the Determination of rAAVs Empty-to-Filled Capsid Ratios

**Figure 1:**
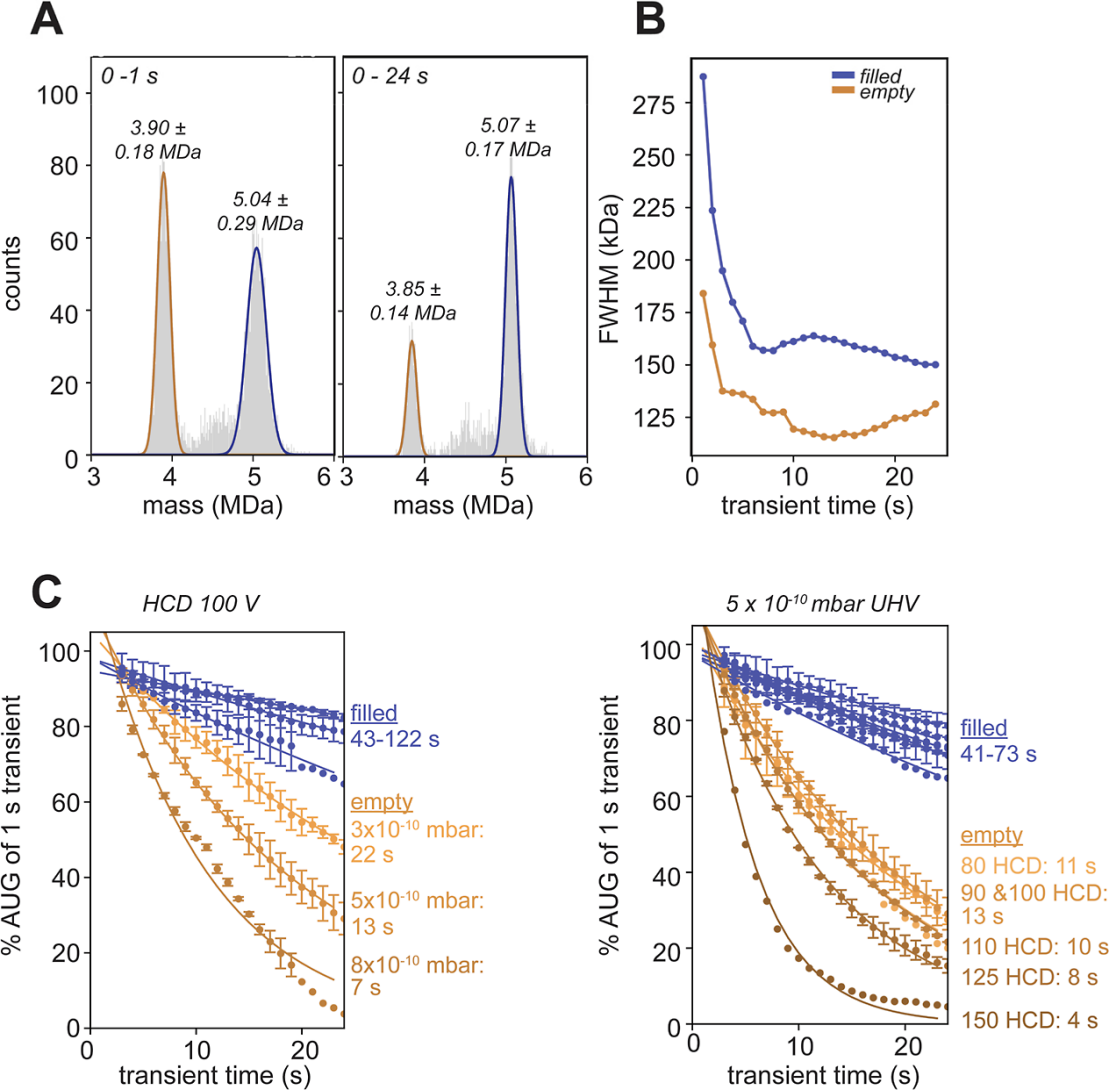
Mass analysis of rAAVs (rAAV9_FP sample I) following ultralong transient acquisition. (A) Extending the analysis from the first 1 s (left) to the full 24 s (right) transient reveals an improvement in mass resolution. However, especially the number of empty capsids that can be traced and analyzed decreases at longer transient times. The mean masses ± FWHM of the empty and filled capsid population are indicated in the graph. (B) When extending the transient time, the empty and filled Gaussian fits show a clear decrease in peak width with a concurrent drop in FWHM. (C) Decline in area-under-Gaussian (AUG) (set at 100% at 1 s into the transient) becomes apparent when assessed over the transient time. Both increased background gas pressure (left) and increased HCD activation (right) affect the ions of the empty capsids. Ions of filled capsids stay relatively stable. The AUG is fitted with a single exponential decay with the ion “half-life times” indicated on the right side. Given is the average percentage with error bars indicating the standard deviation over replicates (for pressure setting *n* = 3, except for 3 × 10^–10^ mbar *n* = 2, HCD80 *n* = 1, HCD90 *n* = 2, HCD100 *n* = 3, HCD110 *n* = 2, HCD125 *n* = 2, and HCD150 *n* = 1).

We initially started to investigate whether long transient times would also benefit the analyses of rAAVs. Therefore, we focused on a sample of rAAVs (serotype 9), which based on MP consisted of a ∼1:1 ratio of empty and filled rAAV9 particles (rAAV9_FP sample I, Figure S1). For native CDMS, we ionized these rAAV particles by static nanoelectrospray ionization and monitored rAAV ion signals while being trapped in the Orbitrap analyzer over the full transient length of 24 s. We segmented the recorded full transients and applied a FT on each subsequent 256 ms segment (termed frequency chasing).^24^ By frequency chasing, we can follow the improvement in mass resolution and sharpening of the mass histograms along the increased transient time (Figure 1A,B). Hundreds to thousands of single ions of rAAVs could be analyzed in this way, most even up to the end of the 24 s recording. This highlights the extraordinary stability of the AAV ion trajectories while traveling extended distances of 100 km in the Orbitrap analyzer, undergoing thousands of collisions with the background gas molecules (Figure S2A). Comparing the full-width-half-maximum (FWHM) attained at 24 s to that of 1 s, a ∼2-fold decrease is observed in both the empty and filled rAAV mass histograms (Figure 1B). The measured FWHM comes ultimately close to the FWHM of the theoretical AAV9 mass distribution (caused by the intrinsic variability in VP1-3 stoichiometries) (Figure S3).^30^ Besides the desired increase in resolution and accompanied increase in peak height of the filled rAAVs, a substantial and undesirable decrease in the number of detected empty rAAVs is observed over the transient time. Crucially, this also results in a change in the determined empty-to-filled ratio (Figure 1A). The mass distribution for the short 1 s transient matches the 1:1 empty-to-filled ratio (determined by the Gaussian fit peak area) observed by MP closely, but at longer transient times up to 24 s the given ratio of 1:2.8 is quite far off (Figures 1 and S1). Notably, upon extended trapping in the Orbitrap analyzer (i.e., with increased transient times), we also observed a marginal but steady decline in the ions’ intensity (Figure S4A). After correcting for this linear decline, the average mass can be inferred for the rAAV subpopulations, which closely agrees with the data obtained by MP (Figures S4B and S1).

To visualize the change in the empty-to-filled ratios over time, we extracted the area under the Gaussian fit (AUG) as observed for the empty and filled particles, normalizing this number to 100% at the shortest analyzed transient of 1 s. The decline in AUG was subsequently determined by extending the analyses of the transient up to 24 s in a 1 s incremental manner. To probe which parameters affect this decline, the analysis was performed at different ultrahigh vacuum (UHV) background gas pressures and different HCD collision energies (Figure 1C). Evidently, both elevated background pressures and higher HCD collision energies led to an increase in ion signal loss, most prominently for ions originating from empty rAAVs (Figure S5). Fitting an exponential decay, we extracted the apparent ion population “half-life times” for the ions originating from empty and filled rAAVs, under all of these different conditions (Figure 1C). These apparent half-life times of filled rAAV particles are in the range of 40–120 s, whereas the population half-life times of the empty rAAV particles are much shorter, in the range of 3–20 s. This indicates that within the Orbitrap analyzer, filled rAAV particles can better survive ultralong trapping times, even well beyond 24 s.

During our analysis, we focused primarily on the empty and “fully” filled particles, but as shown in Figure 1A, the CDMS data also reveals subpopulations of neither empty nor fully filled rAAV particles, not resolved in the MP data (Figure S1). At the best settings for mass resolution (e.g., lowest background pressure of 3 × 10^–10^ mbar and a 24 s long transient), the mass histogram reveals two subpopulations of partially filled capsids (Figure S6). Therein, the capsids seem to contain mostly about 1/2 (53%) or 3/4 (74%) of the intact genome, which we argue may result from genome truncations at the preferred GC-rich hot spots (Figure S6).^7,31,32^

### Extended Trapping of Adeno-Associated Virus Particles within an Orbitrap Mass Analyzer Reveals Frequency Shifts

**Figure 2:**
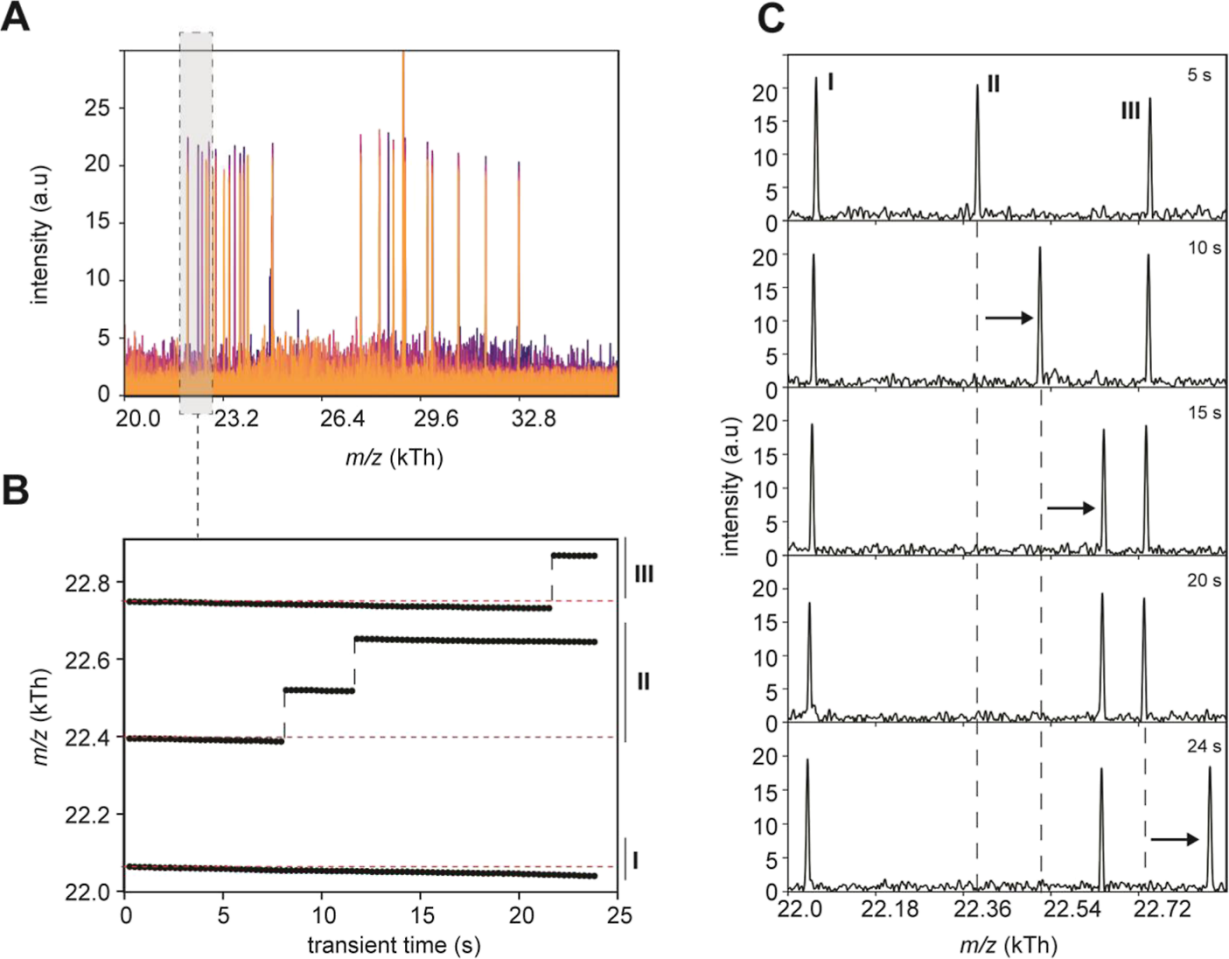
Single CDMS ultralong transient scan reveals distinct rAAV ion behavior. (A) Along the 24 s acquisition, individual rAAV particles can be monitored over the entire transient duration with empty rAAVs in the lower *m*/*z* range and filled rAAVs in the higher *m*/*z* range. The FT signals for different time segments (256 ms) are overlaid with colors ranging from blue to orange. Every spike is a detected single ion of a rAAV particle. (B) Three single-ion trajectories (extracted from the gray window in panel A show a continuous marginal decline in *m*/*z* due to small neutral losses (i.e., desolvation). Incidentally, upward shifts in *m*/*z* hint at a loss of charge by individual ions. (C) Different time segments of the transient reveal the marginal drift to lower *m*/*z*, for ions I, II, and III, and occasional larger jumps to higher *m*/*z*, indicative of a charge loss, for ion II at 10 and 15 s, and for ion III at 24 s.

We suspected that the loss of empty capsids during the ultralong transient recording is due to discontinuous *m*/*z* trajectories, hampering their frequency tracing. For a more detailed investigation into the loss of empty capsids and their *m*/*z* trajectories, we initially focused on a single scan containing several rAAV ion signals. Upon inspection of the single-ion trajectories, we observed that the rAAVs display a gradual loss of mass, resulting in a steady downward drift in *m*/*z* (Figure 2). As reported previously, when recording shorter 1–4 s transients, the origin of this behavior is presumably due to continuous desolvation of the ions, as a result of small neutral losses.^24^ Here, on the extended 24 s time scale, we observed similar gradual desolvation for each analyzed single rAAV ions. Furthermore, while most ions remain stable in their ion trajectory (ion I, Figure 2B,C), several rAAV ions underwent instantaneous, upward shifts in *m*/*z*, sometimes consecutive times (ions II and III, Figure 2B,C). These sudden jumps in frequency are reminiscent of those induced by charge loss, as reported earlier in studies using a linear ion trap, FT-ICR, or Orbitrap analyzer.^23,24,27,33,34^ When evaluating a large number of jumps, a theoretical difference in charge of 1.02 ± 0.02 could be inferred for each jump, assuming the loss in mass is considered negligible on the MDa scale of the analyzed rAAVs (for instance, loss of solvent molecules) (Figure S7). The inferred charge loss is pleasingly close to the loss of a single charge, nominally 1 z. Notably, in the single scan, these charge loss events tended to be much more frequently observed in the lower *m*/*z* range, where the ions originating from the empty capsids are typically measured (Figure 2B,C). Moreover, although less frequent, also quantized jumps were observed with an average difference in charges of 2.03 ± 0.04 and 3.08 ± 0.06, revealing that also sometimes multiple charge species are eliminated at once (Figure S7). The loss of charges, with concurrent shifts in *m*/*z*, profoundly affects the number of particles that can be analyzed, as such ions cannot be traced for the full duration of the extended transient. Together with the loss in particle detection due to ion instability, it can explain the decline in the level of empty particle quantification seen earlier (Figure 1).

### Desolvation of rAAV Ions Seems to Be Reliant on the Cargo

**Figure 3:**
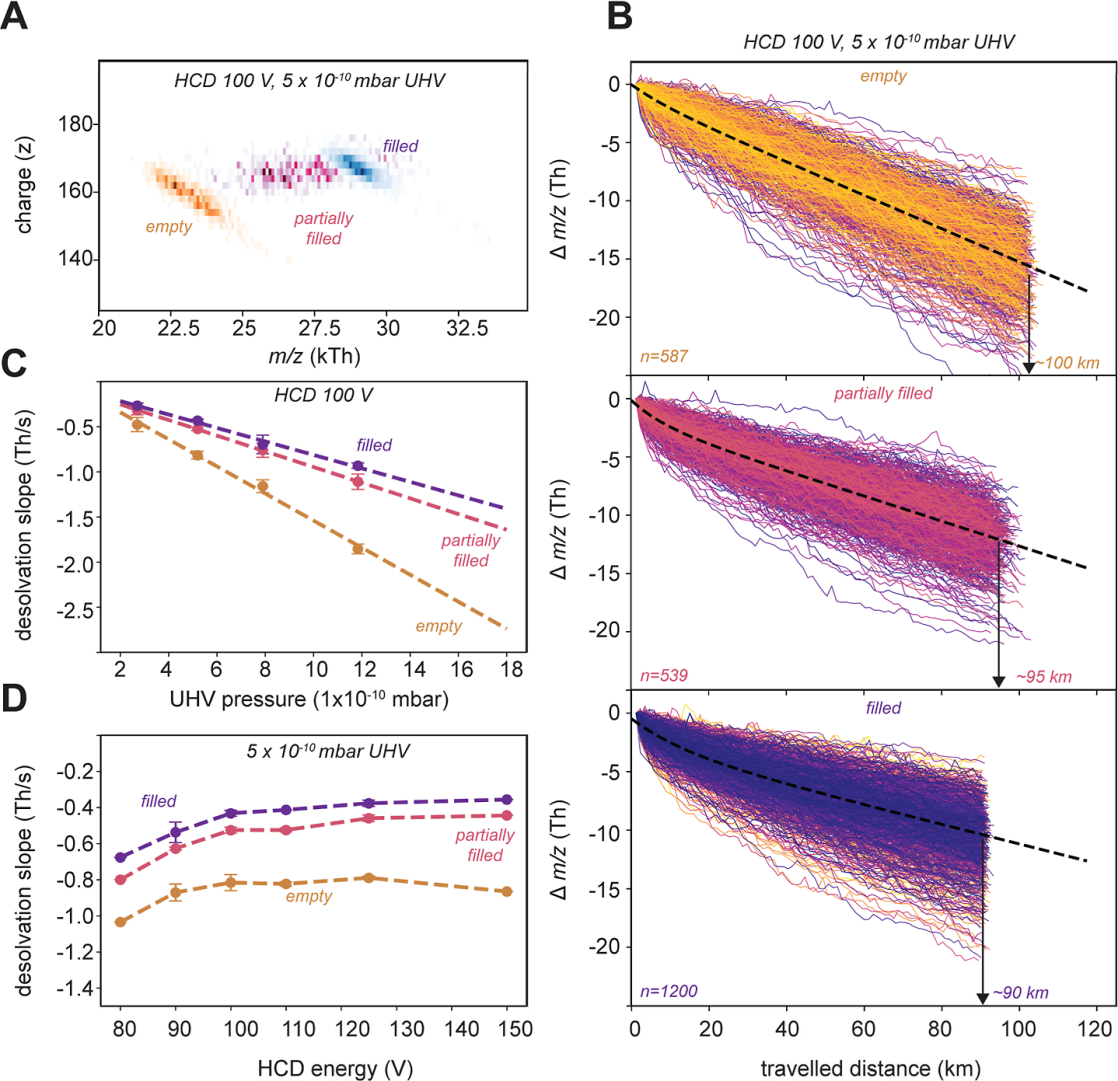
Monitoring the distinct desolvation behavior of rAAV ions during ultralong transients. (A) 2D CDMS histogram of ions displaying stable trajectories. Ions are color-coded based on the absence/presence of (in) complete genetic payload. (B) During ion trapping, desolvation induces a steady decline in *m*/*z*, which is more enhanced for empty rAAVs than the filled particles. The differences in distance traveled between empty (∼100 km) and filled particles (∼90 km) originate from differences in frequency and, thus, velocity of these particles. (C) The desolvation rate is influenced by the pressure in the Orbitrap region. Higher gas pressure results in a stronger desolvation, whereby again, the empty rAAVs are more affected. Slopes of the neutral losses, as seen in Figure S8, were taken of several replicates (*n* = 3, except for 3 × 10^–10^ mbar *n* = 2). (D) Stronger activation of the ions in the HCD cell, prior to entering the Orbitrap analyzer, decreases the observed desolvation rate in the Orbitrap region. The rate of desolvation per second was inferred from the slopes, as shown in Figure S8 (HCD80 *n* = 1, HCD90 *n* = 2, HCD100 *n* = 3, HCD110 *n* = 2, HCD125 *n* = 2, and HCD150 *n* = 1).

The prolonged trapping in the Orbitrap analyzer allows us to monitor rAAV desolvation over extensive periods of time in detail (Figure 2). Therefore, we first traced the rAAV ions, consequently analyzing ions that exhibited no charge losses during the 24 s transient acquisition. For those ions, we constructed a charge versus *m*/*z* 2D histogram (Figure 3A). In this 2D particle histogram, we can readily differentiate the distinct rAAV populations, revealing the presence of empty, partially filled, and fully filled particles (Figure 3A, color-coded). For each subpopulation, we plotted the single *m*/*z* trajectories and monitored the neutral losses over their traveled path (Figure 3B). Therein, we can clearly observe distinct desolvation patterns, where filled and partially filled rAAV particles seem to level off earlier in the desolvation rate, whereas empty rAAVs exhibit a more prominent desolvation rate (Figures 3B and S8). This effect was found to be exacerbated by an increase in the gas pressure in the HCD cell filled with xenon gas. The xenon molecules that diffuse from the HCD cell into the UHV region evidently affect desolvation in the Orbitrap analyzer (Figure 3C). The difference in neutral loss of empty capsids compared with filled capsids can be substantial. To illustrate, at the highest UHV pressure (12 × 10^–10^ mbar in the Orbitrap region), we estimate that the empty capsids lose about 6.5 kDa of mass over a 20 s period compared to ∼ 3.7 kDa for filled rAAVs.

Next to collisions with the background gas molecules in the Orbitrap analyzer, we also found that the collisional activation energy in the HCD cell reduced the desolvation rate of the rAAV ions during mass analysis (Figure 3D). A probable explanation is that higher activation of the ions in the HCD cell results in a more vigorous desolvated rAAV population prior to entering the Orbitrap analyzer, resulting in fewer neutral losses during the recording of the 24 s transients. However, under all tested HCD conditions, the empty rAAV capsid ions maintain a more pronounced loss of solvent molecules compared to the partially filled and filled rAAVs (Figure 3). As recently has been shown that more hydrated ions can display more desolvation in a mass analyzer,^34^ it is tempting to argue that the empty rAAVs remain more hydrated than filled rAAVs in native MS.

### Empty rAAV Capsid Ions Are More Prone to Charge Loss Events

**Figure 4:**
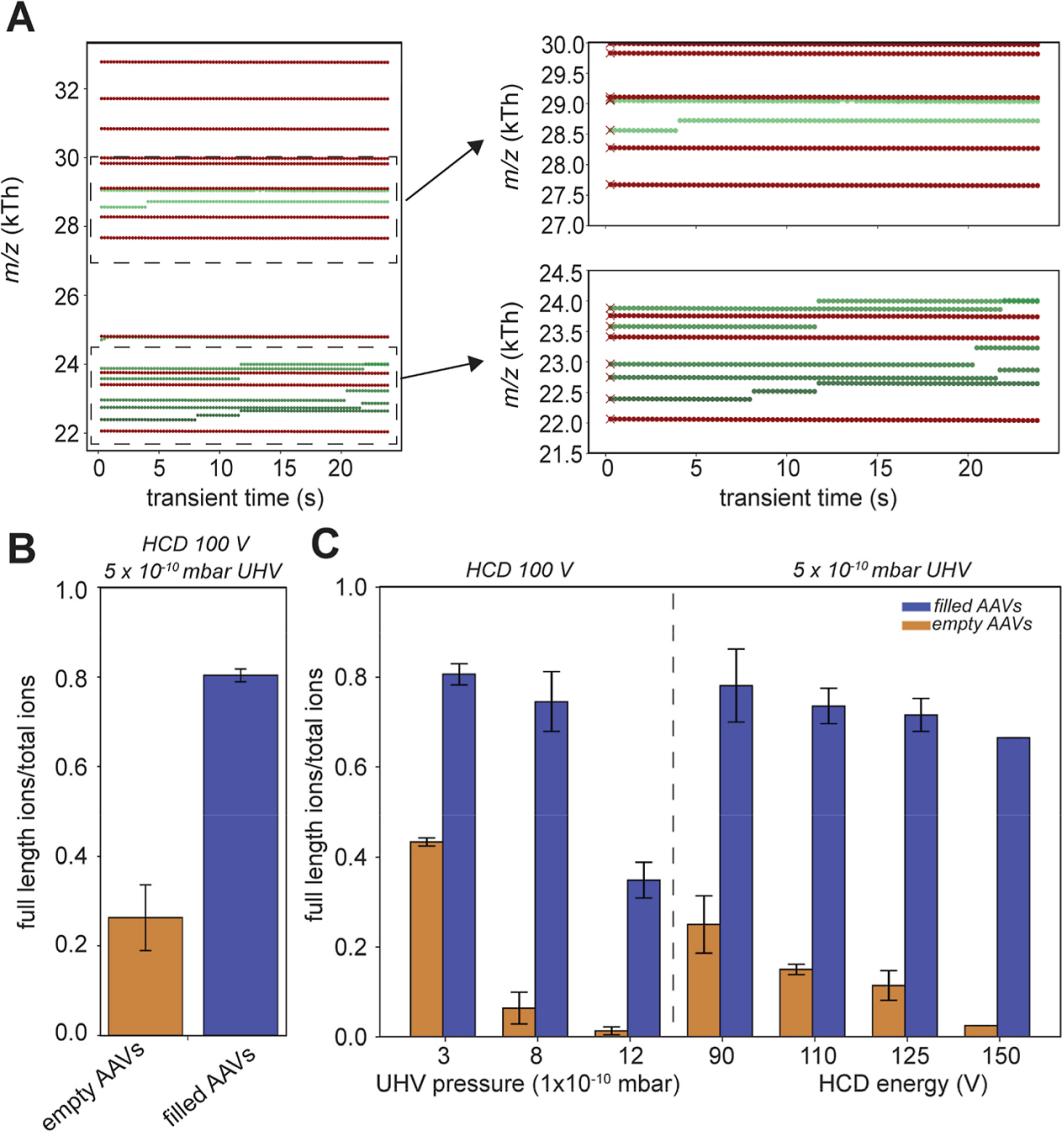
Charge loss events in rAAV ions during ultralong transients. (A) Traces of individual ion frequencies during the 24 s transients. In each scan, the region for empty and filled capsids was monitored for the number of ions that retain a relatively stable trajectory (red) and ions that display charge loss induced frequency jumps (green). (B) Ratio of ions that remain stable from the start to the end of the full-length (24 s) transients with that of all the ions at the start of the transient. A substantial difference in this ratio is observed when chasing ions from empty (orange) and filled rAAV particles (blue). (C) HCD activation and UHV pressure affect the observed ratios. Both increased collision energy and gas pressure appear most detrimental to the trajectories of empty rAAVs (different gas pressures *n* = 3, except for 3 × 10^–10^ mbar *n* = 2, different HCD levels *n* = 2, except for HCD100 *n* = 3, and HCD150 *n* = 1).

We next focused on the observed charge loss events (Figures 2 and S7). By clustering the ion trajectories in each scan, we classified the single ions that remained stable versus the ions that had discontinuous trajectories, exhibiting precise “jumps” in *m*/*z* (Figure 4A). In doing so, we observed that the empty rAAV capsid ions underwent substantially more “jumps” compared to filled capsid ions (Figure 4B). Setting a higher gas pressure in the HCD cell increased the number of ions that undergo a frequency shift during the transient. Especially for the ions originating from the empty rAAVs, such higher pressures had a detrimental effect. At the highest pressure (12 × 10^–10^ mbar UHV), most of the empty capsids (∼99%) experience at least one break during the 24 s transients. Although, at this point, a complete loss of ion detection can also be seen for some particles (Figure S9). Notably, stronger activation in the HCD cell with an elevated collision energy also translates into an increase in the frequency shifts of empty capsids (Figure 4C). Ultimately, with charge losses dominating the ion trajectories of empty capsids, it becomes challenging to trace the rAAV particles over the transient time, leading to an unwanted bias in the quantification of mainly empty rAAVs. This observed difference in behavior of ions of empty and filled rAAVs is critical for the use of Orbitrap-based CDMS for assessing empty-to-filled ratios of rAAVs, and has, so far as we know, not been taken into account in the numerous reports from others and our group.^6,7,13,17,19^ Although the effect is limited to short transient times, the loss of empty capsids can be substantial. Therefore, we next examined whether we could potentially avoid or resolve this issue.

### Ion Trajectories Can Be Stabilized by Chemically Induced Charge-Reduction

**Figure 5:**
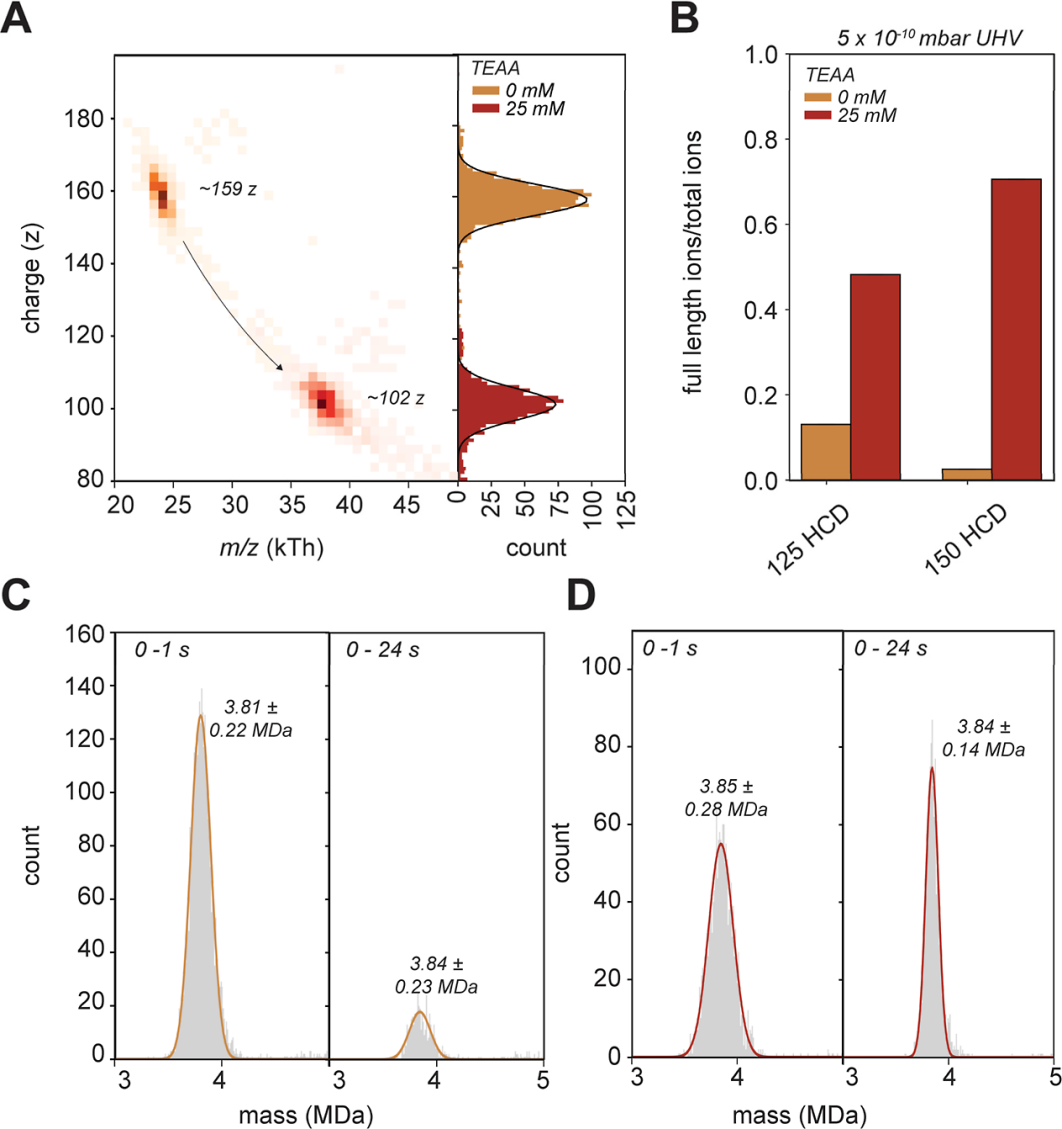
CDMS analysis of charge reduced empty rAAVs. (A) Overlay of 2D histogram displaying ions of noncharge reduced (orange) and charge-reduced (red) rAAVs, measured separately. Charge reduction shifts the ions from 159 z and 24 kTh to about 102 z and 37.5 kTh. Charge histograms are displayed on the right side. (B) Charge reduction of empty capsids with TEAA restores substantially the ions’ stable trajectories. (C) Mass histograms of the empty rAAV capsids measured in the absence of TEAA and HCD set at 150 V. Due to the extensive number of charge losses, the population of ions originating from empty capsids drops dramatically with an increase in the transient time, from 3096 to 442 counts. (D) Mass histograms of empty rAAV charge reduced by using TEAA. The number of jumps is substantially reduced and the total ion count remains in the same order (from 1625 to 1179 counts). Meanwhile, the mass resolution still benefits from the 24 s transient.

Because collisions with the background gas most likely trigger the charge loss events of the empty capsids (these collisions also possess a higher energy transfer compared to filled, Figure S2B) and concomitantly impair the frequency chasing and quantification of these rAAVs, we next focused on minimizing the ions’ velocity in the Orbitrap analyzer. For this purpose, we used the charge-reducing agent triethylammonium acetate (TEAA) as an additive during the electrospray ionization process. Empty capsids of the same AAV serotype (AAV9) were ionized with or without 25 mM TEAA and analyzed using the 24 s transients. We hypothesized that a TEAA-induced charge reduction would lower the frequency and ion velocity of the empty rAAVs into a similar *m*/*z* range as filled rAAVs without charge reduction. Indeed, without TEAA, the empty particles are observed with charges between 155 and 165, centered around *m*/*z* 24000, whereas with the addition of TEAA, this changes to a z-range of between 90 and 110, centered around *m*/*z* 37500 (Figure 5A). The reduction in charge led to a substantial decrease in the number of charge loss events (Figure 5B), indicating that the ion velocity and traveled path are key parameters that affect the number of observed frequency jumps. To compensate for the lower energy with which the charge-reduced capsids enter the HCD cell, we also measured them at a higher HCD setting of 150 V. Also, an improvement of the number of stable ions can be seen here (Figure 5B). Whereas initially, without using the charge-reducing agent, we lost ∼85% of the empty rAAV ions after 24 s (Figure 5C), there is seemingly no reduction in traceable particles of empty rAAVs following charge reduction by using TEAA (Figure 5D). Also, now enabling the full 24 s transient for particle tracing, charge reduction provides a higher resolution mass histogram (compare Figure 5C,D). Therefore, the use of TEAA opens the possibility to recover reliable quantification of rAAV particles, at the beneficial ultralong transient-improved mass resolution.

### Charge-Reduction Facilitates Quantification of rAAVs Following Long Transient CDMS

**Figure 6:**
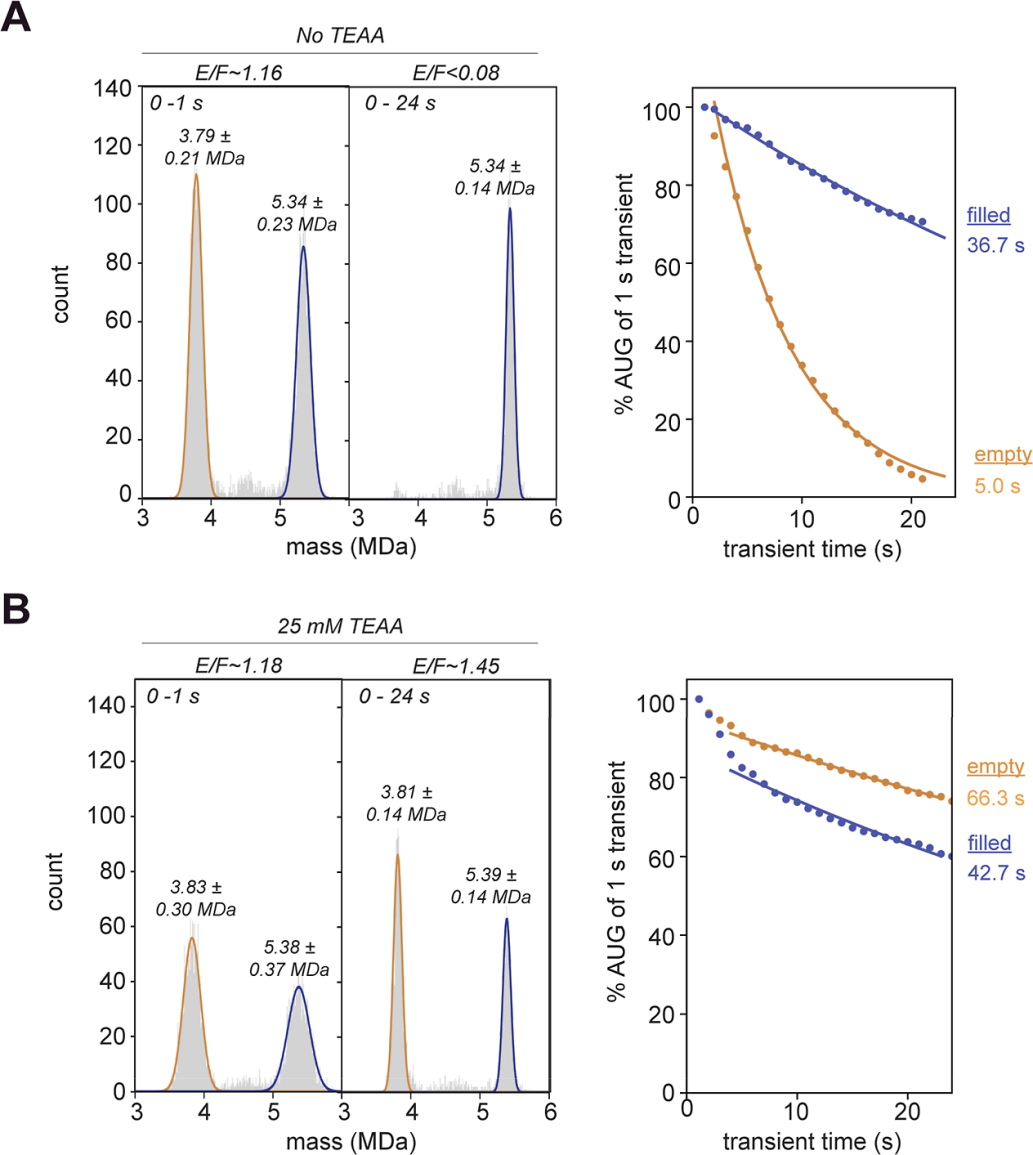
CDMS analysis of empty and filled rAAV (rAAV9_FP sample II) with or without charge reduction. (A) A second sample of rAAV9s (with both empty and filled capsids) was measured in the absence of 25 mM TEAA. Without charge reduction, the empty capsids contain nonideal *m*/*z* trajectories and almost none of them can be traced over the full 24 s transient (left). The decline in analyzed empty capsids can be seen over accumulative transient times (right). The calculated ‘half-life times’ are annotated next to the graph. (B) The same analysis was performed on a sample with charge reduction by 25 mM TEAA. The empty capsid population remains stable in the mass analyzer and can now be traced resulting in an accurate mass analysis (left). This can also be seen when plotting the AUG of the fitted mass histograms over accumulative transient times (right). In the mass histograms, the average mass ± FWHM is indicated per particle population.

The charge reduction strategy, initially demonstrated on a sample just containing empty AAVs, was validated on a second AAV9 sample containing close to 50% empty and 50% filled particles (Figure S1). This sample was measured by long transient CDMS up to 24 s, either in the presence of 25 mM TEAA or without TEAA. As seen earlier, most filled particles were retained in the Orbitrap mass analyzer and remained stable in their *m*/*z* trajectory, regardless of the number of charges. After a 24 s transient, the observed mass peak of the filled population was about 5.3 MDa and contained a peak width of about 136 kDa FWHM (Figure 6A). In contrast, the presence of TEAA markedly influenced the empty capsids. In the absence of the charge-reduction agent, the empty particles are not traceable anymore after the 24 s transient recording, with only a few empty capsids left that could be traced and plotted in the mass histogram. Because the empty capsid detectability is strongly affected, no mass information can be obtained from this measurement. The particularly strong decline in empty particles seen here can reflect batch-to-batch variability in measuring rAAVs as a relatively high UHV pressure of 7.8 × 10^–10^ mbar is needed to transmit the high-mass filled particles (∼5.3 MDa). Applying 25 mM TEAA to the sample essentially restores the empty/filled capsid ratio, with about 60 to 80% of the empty and filled particles maintaining their trajectory during the transient recording (Figure 6B). We can now infer an average mass for the empty capsids of 3.81 MDa with a peak width of 143 kDa of the FWHM. Both empty and filled particles still display a minor loss of ions during trapping. However, this time, the rAAVs have similar decay rates in the 40 to 70 s range. Intriguingly, it is early in the transient that some filled capsids tend to decay more quickly. Potentially, the relatively high UHV pressure applied for good transmission of the filled particles might have a downstream effect on their survival in the mass analyzer. In the end, an empty/filled ratio of 1.45 is extracted following charge reduction, close to the 1.12 ratio obtained by MP (Figure S1). Thus, by employing charge reduction, the quantification is much closer to the expected value, while at the same time we achieved a high-resolution CDMS measurement.

## Discussion

Over the past few years, CDMS has become an important tool for the analysis of the rAAV-based gene therapy products, especially for the quality control of the capsid integrity and for assessing the effectiveness of packaging the transgene during the production process. Such CQAs are hard to assess due to the high mass of the particles, the heterogeneous nature of their genome-cargo, and the stochastic nature of the capsid assembly. Ideally, quality control provides baseline separation of the different co-occurring particles in a manufactured therapeutic batch. This requires high-resolution power (i.e., mass resolution) when the rAAVs are separated by the molecular weight. Recording longer transients in Orbitrap-based CDMS leads to a higher mass resolving power and improved signal-to-noise, potentially enabling the better determination and quantification of all co-occurring empty, partially filled, and filled particles. The data presented here clearly demonstrate that trapping and analyzing ions, originating from empty and filled rAAVs, for more than 20 s in the Orbitrap during CDMS, indeed leads to a higher resolving power and an enhanced ability to distinguish the rAAV populations. However, we find that this comes at the expense of an undesired decrease in the determined empty-to-filled ratio, a CQA of therapeutic rAAVs. We show that this unwanted artifact is predominantly caused by the relatively unstable ion trajectories of the ions originating from empty rAAvs particles due to ion desolvation and charge losses. These latter events are way more dominant for empty rAAvs than filled rAAVs, and their occurrence increases not only with longer transients but also with a higher background pressure in the Orbitrap analyzer. We argue that the numerous collisions with the background gas during the trapping in the Orbitrap analyzer cause these phenomena, arguing that the ions of empty rAAVs undergo more and higher energy collisions than those of filled rAAVs. Based on that hypothesis, we analyzed the behavior of charge-reduced rAAV ions using TEAA as an additive in the electrospray solution. This decreased the average charge of the ions from ∼160 to ∼100 and also lowered their frequency and velocity in the trap (thus being detected at higher *m*/*z*). The charge reduction resolved any issues originating from charge losses, and we observed that nearly all ions of empty rAAVs retained their stable ion trajectories over the full 24 s. This further indicates that the relatively higher energy collisions with the background gas during the longer trajectories may have caused the ion losses observed using noncharge-reduced native CDMS. Therefore, charge reduction seems to provide an attractive way to enable extended transient recording times while preserving the accurate assessment of empty-to-filled ratios by Orbitrap-based CDMS. The effects on ion trajectories, as observed in our analysis, should be taken into consideration while performing CDMS of rAAVs and other macromolecular complexes, especially in quantitative analyses. When properly addressed, Orbitrap-based CDMS can be used as a precise tool to mass analyze a variety of AAV particles and obtain an accurate assessment of CQAs, such as the relative contribution of over- or underfilled particles (e.g., by truncated genomes) and the quantitative analysis of empty-to-filled ratios.

## Supporting Information Available

Experimental materials and methods; AAV MP analysis; calculated theoretical collisional energies of empty, filled, and charge-reduced AAVs; simulated AAV9 mass distribution; analysis and correction of intensity drifts for measured AAVs; differential ion decay empty versus filled AAVs; potential AAV genome truncations; inferred charge losses; neutral losses of empty, partially filled and filled AAVs over transient time; and loss of ion detection during transient (PDF)
