# Supplementary material for: Prolonged Trapping of Adeno-Associated Virus Capsids Reveals that Genome Packaging Affects Single-Ion Mass Spectrometry Measurements
Source: J Am Chem Soc. 2025 Mar 24;147(13):10925–34. doi: 10.1021/jacs.4c13393 (PMC11969545; doi:10.1021/jacs.4c13393)
Supplement: Supplementary file 1 — ja4c13393_si_001.pdf [file ja4c13393_si_001.pdf]

Supplementary Information: Experimental Methods and Figures

Prolonged trapping of adeno-associated virus capsids reveals that genome packaging affects single-ion mass spectrometry measurements

Eduard H.T.M. Ebberink[1,2], Victor C. Yin[1,2], Evolène Deslignière[1,2], Arjan Barendregt[1,2], Tobias P. Wörner[3], Kyle L. Fort[1,2,3], Alexander A. Makarov[1,2,3] and Albert J.R. Heck[1,2]

[1] Biomolecular Mass Spectrometry and Proteomics, Bijvoet Center for Biomolecular Research and Utrecht Institute for Pharmaceutical Sciences, University of Utrecht, Padualaan 8, 3584 CH Utrecht, The Netherlands

[2] Netherlands Proteomics Center, Padualaan 8, 3584 CH Utrecht, The Netherlands

[3]Thermo Fisher Scientific (Bremen) GmbH, Bremen, Germany

Supplementary Methods

MS sample preparation

The rAAV stock solutions were prepared for MS measurement as described in detail elsewhere.[1] Briefly, the different rAAV samples were transferred to a 75 mM ammonium acetate solution using a 40-kDa MW limit Bio-Rad P-30 Micro Bio-Spin column, following vendor recommendations. Of the buffer-exchanged stock, about 3 µL of solution was loaded into a gold-coated borosilicate capillary (prepared in-house) for nano-electrospray ionization. Following electrospray, rAAVs were measured on an Thermo Scientific Q Exactive UHMR Orbitrap mass spectrometer in positive mode.

MS parameters and instrumentation for ultralong transient CDMS

The calibration of the UHMR in *m/z* was performed using cesium iodide clusters in the range between 350 and 12,000 *m/z*. For the CDMS measurements of rAAVs, the following transmission parameters were used: capillary temperature 250 °C, injection flatapole, inter-flatapole lens, bent flatapole and transfer multipole, 10, 10, 4 and 4 V respectively. The in-source trapping was set at -50 V for rAAV9_FP sample I and -10 V for rAAV9_FP sample II. The injection time varied between 1 and 500 ms. In all measurements, Xenon gas was used as collision gas, and typical measurement times ranged from 30 to 60 minutes. For the rAAV9_FP sample II an UHV pressure of 7.8×10^{-10} mbar was applied and an HCD energy of 125 V and 200 V for the non-charged reduced and charge reduced measurements, respectively. To apply the ultralong transient, we installed an FTMS Booster X2 (Spectroswiss) to record data externally and independently of the onboard UHMR electronics as described previously.[2] With the FTMS Booster X2 firmware, the final time-domain sampling frequency was set at 390625 Hz, corresponding to an effective low-*m/z* cutoff of 2795 Th. As previously reported, a custom ion pulsing schema, including buffer scans to stabilize the on-board electronics, was prepared to facilitate the ultralong transient measurements on the UHMR instrument.[2]

Ultralong transient signal processing

Raw signals from the FTMS Booster X2 recordings were first processed using Peak-by-Peak version 2023.3.1 (Spectroswiss). The resulting time-domain transient data files were subsequently processed using a combination of Peak-by-Peak and in-house developed Python scripts, a 'frequency-chasing' approach was applied much in accordance with earlier reports.[2, 3] Briefly, an mFT was used with three times zero-filling and apodization using a full-window Hamming function to convert the segmented transient data from the time domain to the frequency and *m/z* domain. Centroids of the *m/z* peaks were calculated for each time segment to perform single ion frequency chasing. Single ion trajectories that were not consistent in *m/z* and thus 'unstable' were removed (by applying a conditional statement if the rolling standard deviation of the last four *m/z* values > 2) from further mass analysis. Signal intensity to charge conversion was done based on a calibration with GroEL as reported previously.[2] To delineate the different rAAV subpopulations (i.e., empty, partially filled and filled) we applied a Gaussian mixed model clustering in the 2D space of charge and *m/z*-value. For the binning of rAAVs in the 2D-histogram of Figure 3, we used a 2 z bin width in charge and 100 Th bin width in *m/z*. For the mass histograms we used a 10 kDa bin width in mass.

Following classifying the ions in empty, partially filled or filled rAAVs, individual *m/z* and intensity paths were extracted to analyze the desolvation (i.e., Δ *m/z*), intensity drift and travelled distances over the

full duration of the transient. The drift in *m/z* was fitted using the following curve: A*exp(B*t) + C*t + D. The desolvation rate was determined by the linear term of the fit (Fig. S8). The intensity drift was characterized by fitting a linear curve, the fitted curve was also used to correct the intensity drift when preparing the mass histograms. Mass histograms with uncorrected intensity drifts were used for tracking the peak widths and area under the Gaussian fit (AUG) over the transient time (Fig. 1 and 6). Because of extensive charge losses, not all ions can be traced to 24 seconds, especially when a high background pressure or HCD energy is used. In that case, the neutral losses were characterized up till an appropriate time point. The calculation of the travelled distance is primarily based on the ion's frequency in the Orbitrap analyzer and together with the calculation of their kinetic energy and energy transfer per collision have been described previously.[2, 3]

The frequency jumps were characterized by evaluating each single scan and applying a 1D clustering algorithm on the *m/z* trajectories.[4] Clusters were then examined to either contain full-length ion paths (no jump) or broken ion paths (jump). Within one measurement (containing numerous scans) and within specific *m/z* regions (low *versus* high *m/z*), the number of ions that displayed no jump was divided by the number of ions at the start of a scan. To infer the difference in charge following a frequency jump, we used the average charge based on the corrected intensities (Fig. S4 and S7).

Supplementary Figures

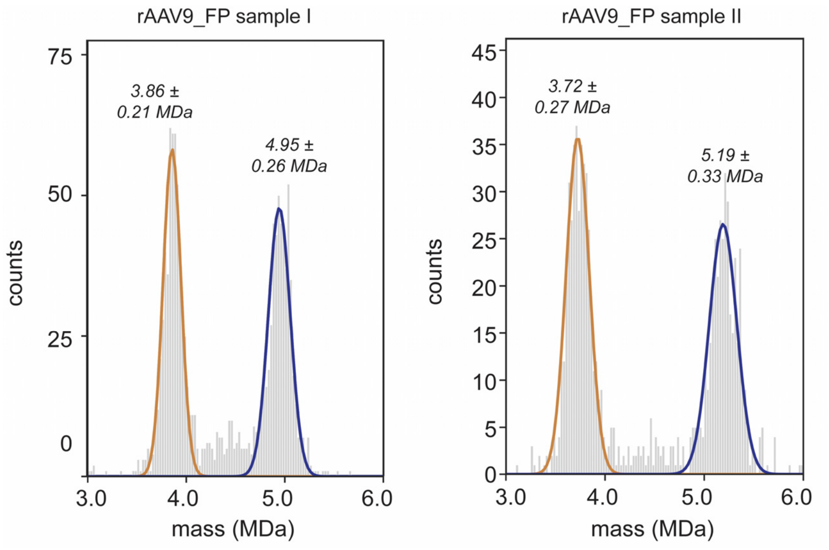

Figure S1: Mass photometry analysis of the two different rAAV9 samples used in this work. The rAAV9 samples were prepared with a genome that can transcribe for a fluorescent protein in both cases (with in sample II a somewhat larger gene element). Both preparations resulted in about a 1:1 empty and filled capsid ratio as here also verified by mass photometry. The mass histograms were constructed with a 25 kDa bin width. Indicated are the average masses ± FWHM of the subpopulations. For the rAAV9_FP sample I a minor element of partially filled particles can also be seen. The empty-to-filled ratio based on the area under the curve of the Gaussian fits shown here (solid lines) was 0.99 for rAAV9_FP sample I and 1.12 for rAAV9_FP sample II. Mass photometry measurements and analysis were performed as previously described in detail.[1]

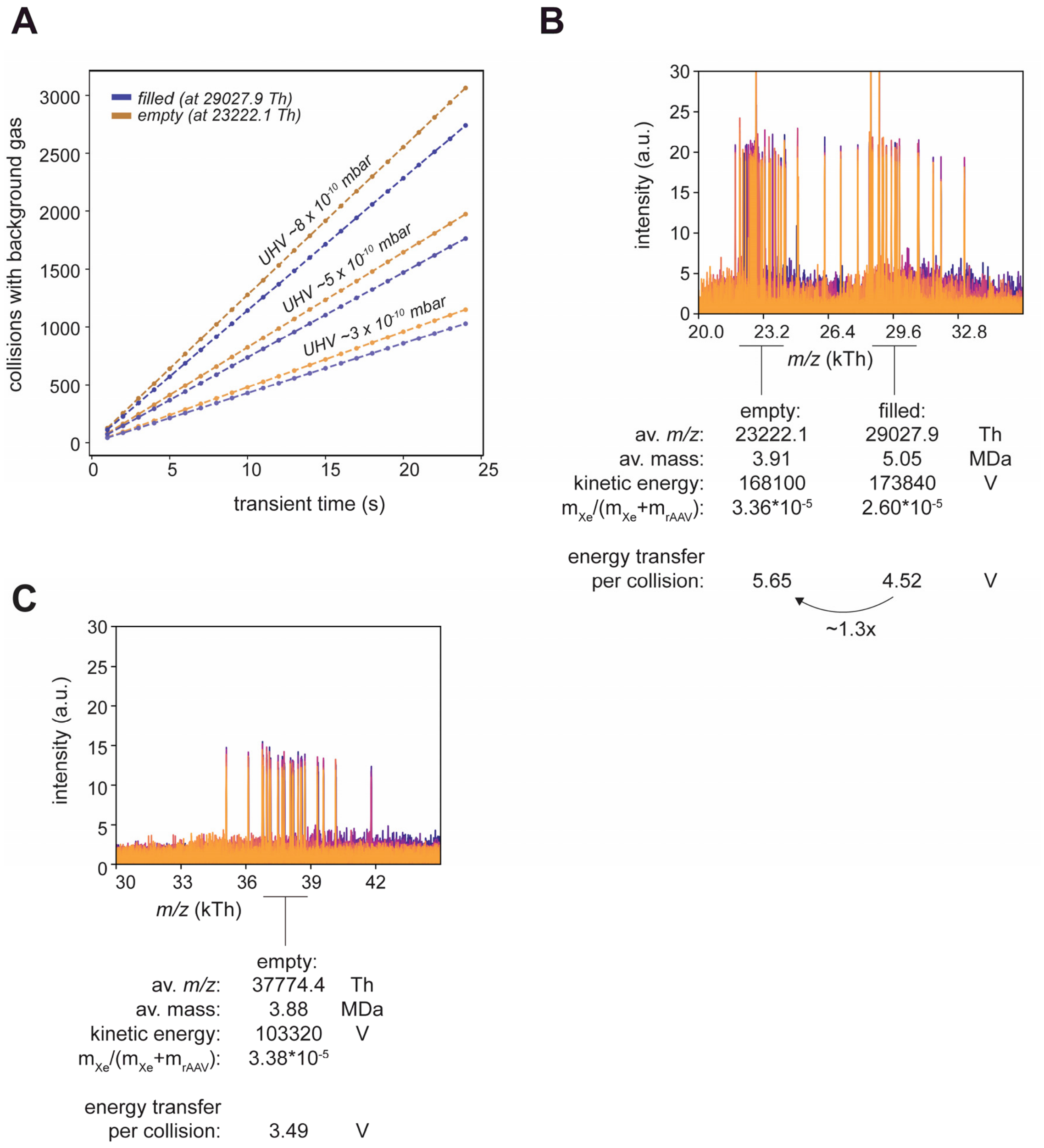

Figure S2: Comparison of theoretical number of collisions and collisional energies for empty and filled capsids. **A)** Based on the frequencies, the theoretical number of collisions was calculated for empty (orange) and filled (blue) capsids with the background gas at different pressure settings. The increase in background pressure introduces more collisions for empty than for filled capsids. **B** & **C)** For calculation of the transferred energy per collision we looked at the average *m/z*, intensity values and masses. Shown is an exemplary scan with empty and filled rAAVs showing the typical spikes in the spectra for each particle, with below indicated the different ion populations of empty and filled capsids. Every color represents an overlay of the time segments analyzed by FT. Accordingly, empty capsids attain more energy per collision than filled capsids, 5.65 and 4.52 V, respectively. **C)** For empty capsids that are charge reduced with 25 mM TEAA the estimated collision energy per collision is substantially lower due to their lower frequency and thus kinetic energy, namely 3.49 V.

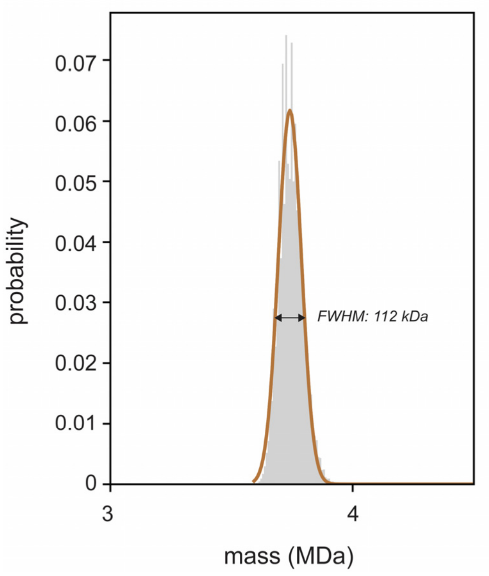

Figure S3: Simulation of the mass distribution for AAV9. Based on the VP stoichiometry of this sample we calculated a theoretical mass distribution of AAVs. For this simulation we used a VP1:VP2:VP3 subunit expression ratio of 8.8:10.9:80.3 as determined by intact protein LCMS of the same sample. The FWHM of about 0.11 MDa is close to the FWHM seen in our measurements with the extended transient times.

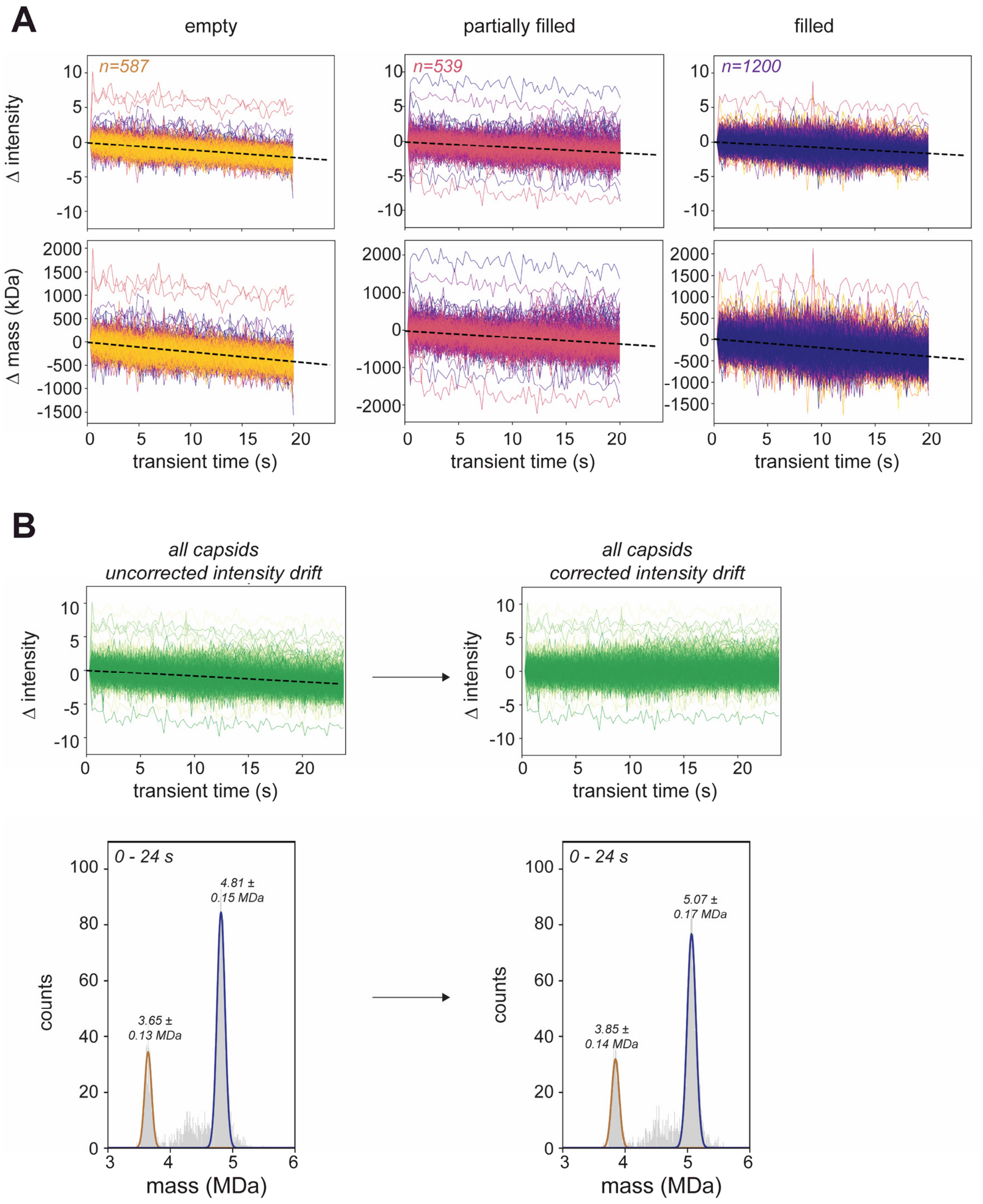

Figure S4: Single ion intensity drifts affect mass accuracy but can be accounted for. **A)** The intensity and deconvoluted mass values were traced for every single ion of rAAV that does not show a frequency jump over the full transient time. The rAAV particles were categorized as empty, partially filled or filled based on their average charge and *m/z*. The steady linear declining drift in intensity (top) affects the deconvoluted mass by resulting in a similar mass drift (bottom). In each box, the number of single ions traced is indicated. Note that the decline in intensity and mass is similar for all categories of rAAV particles. **B)** Following the linear fitting of the intensity drift (using all ions: empty, partially filled and filled), the intensity values were corrected (right top). Following intensity drift correction mass histograms were generated (bottom). Correction for the drift also improves the mass assignment, bringing those close to the masses observed by mass photometry (Figure S1).

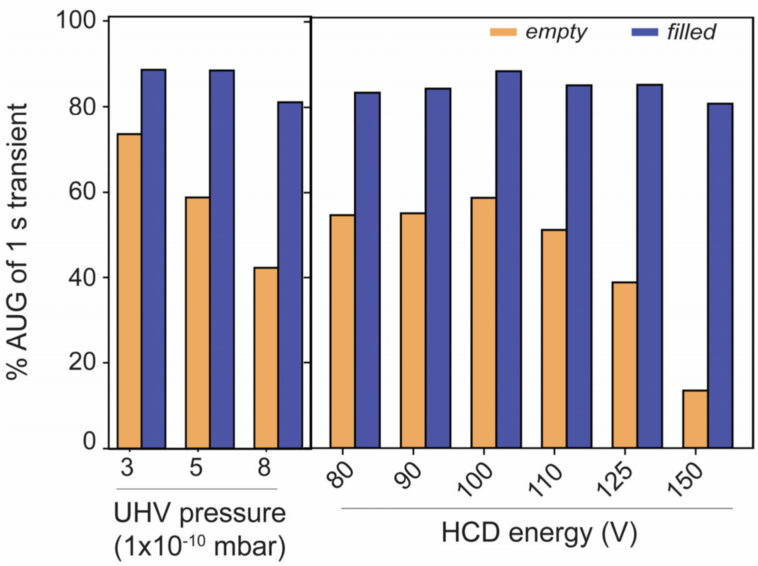

Figure S5: Decline in the number of rAAV particles analysable after 12 s of the transient compared to 1 s. The area under the Gaussian fit (AUG) at 12 s was compared to that of 1 s (set at 100%). While filled capsids (blue) show a modest decline in the number of particles contributing to the mass analysis, the number of empty capsids (orange) processed for their mass declined substantially. An increase in background pressure or HCD energy elevates this decline, again especially for empty rAAVs. This indicates that empty particles are more affected by these transmission settings and are more rapidly removed from mass analysis.

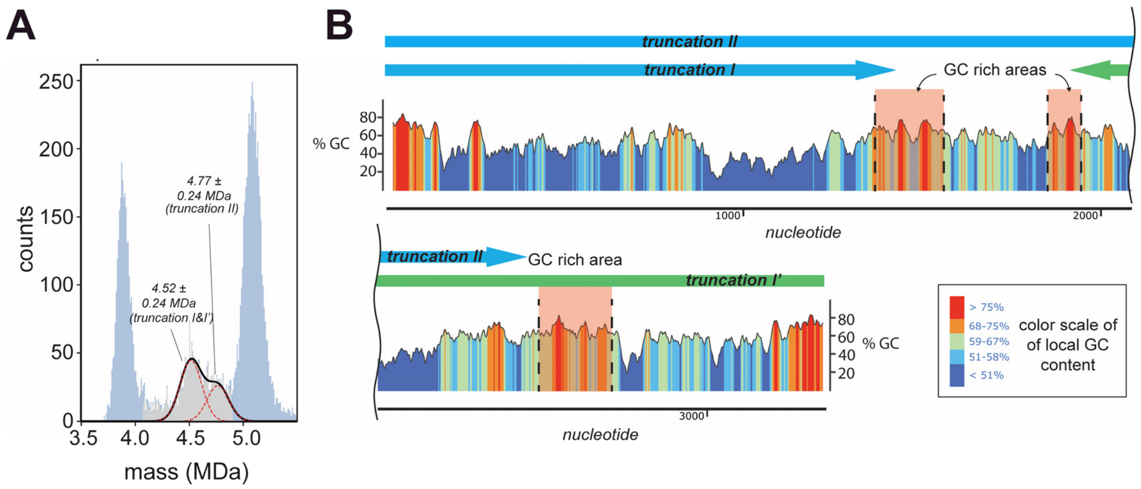

Figure S6: Genome truncations within guanine-cytosine (GC) rich regions may be the cause of partially filled capsids of rAAV9_FP sample I. **A)** With the improved mass resolution, two subpopulations of partially filled capsids (in grey) could be fitted with a Gaussian curve at about ½ and ¾ of the genome size (difference between empty and filled population, displayed in blue). **B)** The CMV-GFP transgene contains several GC-rich regions that may form secondary structures. The percentage GC content was calculated using SnapGene with the formula sum(G or C)/sum(A,T,G or C) for a sliding window of 25 nucleotides. A high percentage of GC content (> 75% indicated in red) is located roughly halfway and at three-quarters of the genome indicated by the shaded areas. The potential formation of secondary structures at those sites might be a cause for the ½ and ¾ filled capsids as detected in the mass histogram.

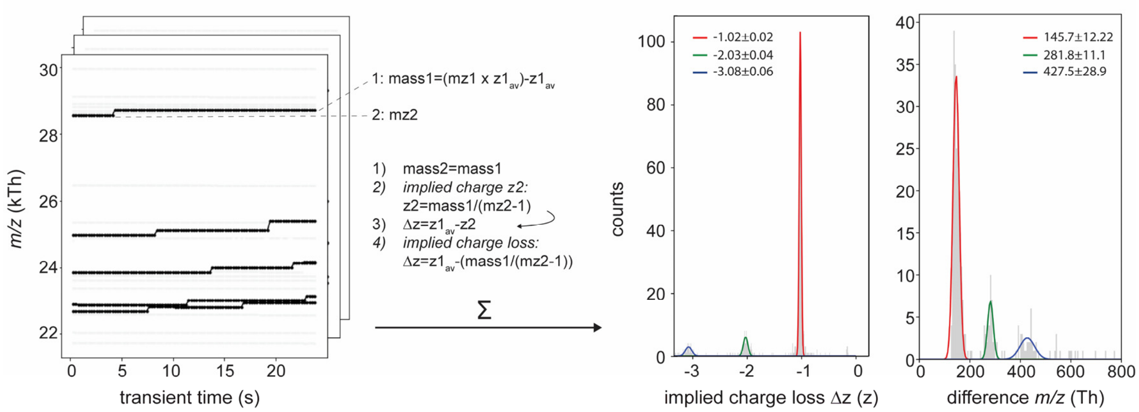

Figure S7: Charge losses inferred for the single ion frequency jumps. Throughout the measured long-transient scans single ion frequency jumps (and thus shifts in *m/z*) are regularly observed (left). For each jump, the implied Δz could be calculated using the depicted formulas (middle). For the assessment of the mass (mass1), the average charge ($z1_{av}$) of all the datapoints after the jump were taken, for the *m/z* values (mz1 and mz2) the datapoint before or after the jump were taken. Under the assumption that for each jump the mass of the ion stays nearly equal (mass2=mass1), the Δz was implied. All the determined Δz were accumulated in a histogram (right). Depicted is also a histogram binning the *m/z* difference for each jump. The inferred charge losses are expected to be quantized integer numbers. Indeed, the jumps correlate closely to an integer number of 1 (1.02±0.02), but also jumps of 2 (2.03± 0.04) and 3 (3.08± 0.06) charges seem to occur.

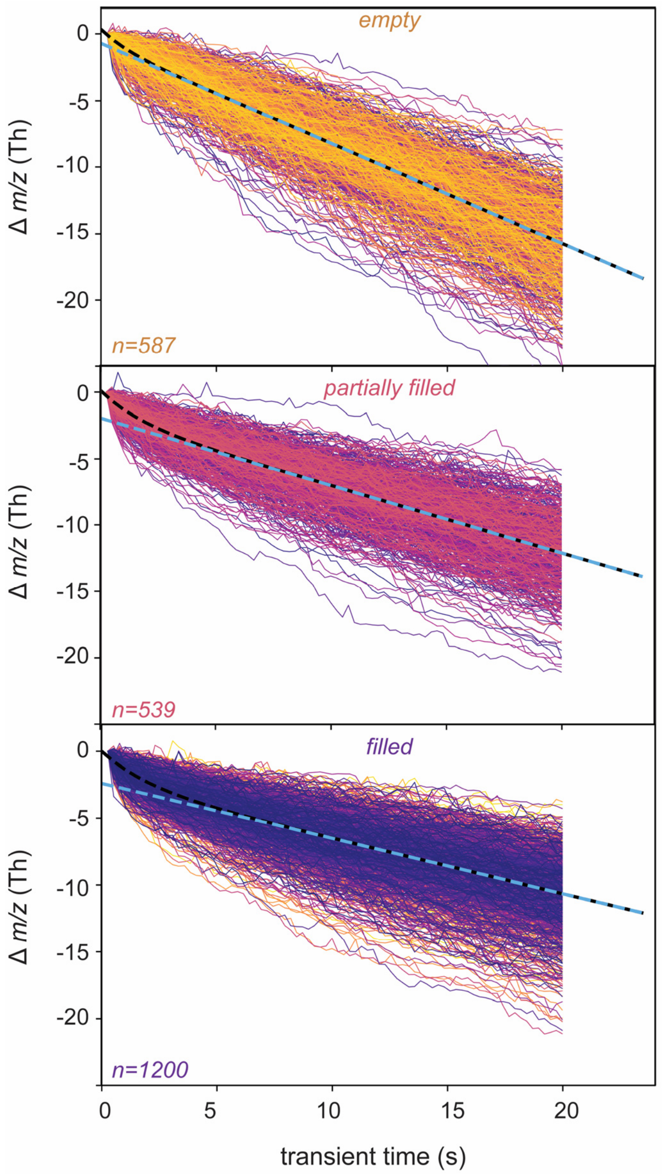

Figure S8: Neutral loss traced over the transient time. For the different rAAVs subsets of rAAV9_FP sample I (empty, partially filled and filled) the neutral loss was traced over the transient time. Indicated are the subset type and number of particles analyzed. The empty particles lose more mass due to desolvation than the filled particles. Shown here are the *m/z* traces up to 20 s for an ultralong transient measurement done at 5 x 10^{-10} mbar UHV and HCD energy of 100 V (see Figure 3). The desolvation rate was derived from the slope of the linear term (blue dashed line).

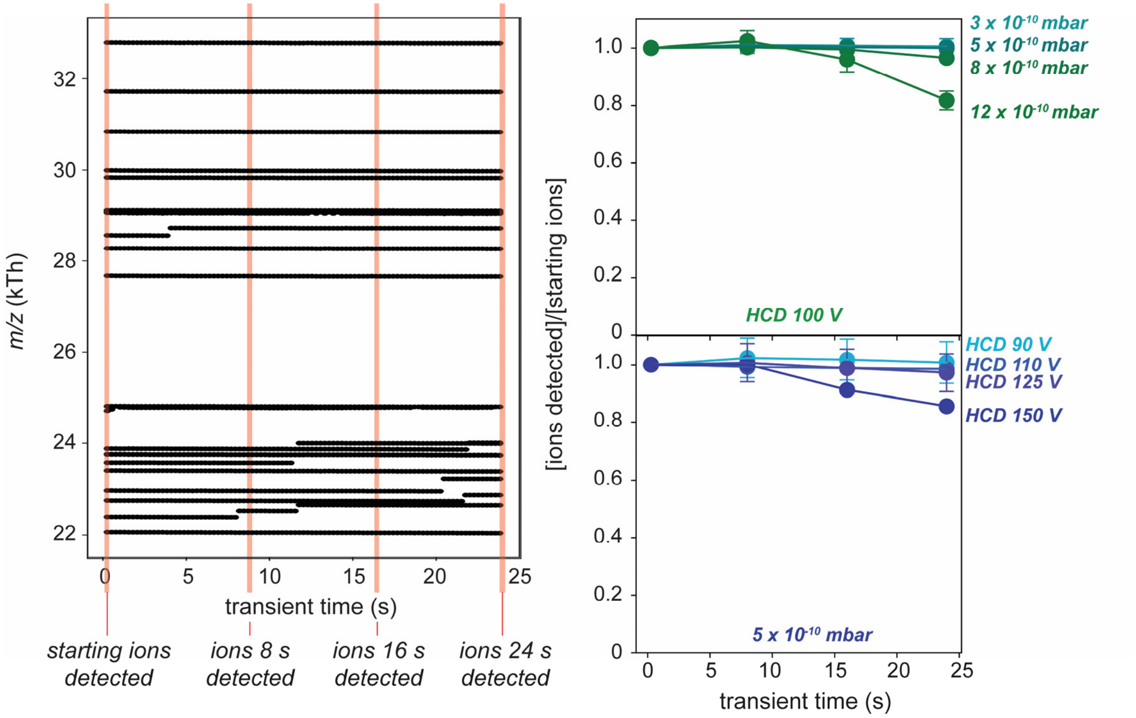

Figure S9: Loss of ion-detection over the 24-second transient observed for the rAAV9_FP sample I. A substantial part of the ions experience charge losses with accompanying shifts in *m/z*, however some ions can also be completely lost from detection. To probe the complete loss of ion detection, we sampled the number of ions at specific time points during the transient recording (0.256, 8, 16 and 24 seconds) (left). For the different measurements we plot the ratio of detected ions divided by the number of ions at the start (~0.256 s) of the transient (right). Only, at the more extreme conditions of high pressure and/or high HCD energy, some ions are lost and cannot be detected anymore in the Orbitrap analyzer at later timepoints. Displayed are the weighted (by ion count at the start) averages with error bars indicating the weighted standard deviation.

Supplemental References

1. Ebberink, E.; Ruisinger, A.; Nuebel, M.; Thomann, M.; Heck, A. J. R., Assessing production variability in empty and filled adeno-associated viruses by single molecule mass analyses. *Mol Ther Methods Clin Dev* **2022,** *27*, 491-501.
2. Desligniere, E.; Yin, V. C.; Ebberink, E.; Rolland, A. D.; Barendregt, A.; Worner, T. P.; Nagornov, K. O.; Kozhinov, A. N.; Fort, K. L.; Tsybin, Y. O.; Makarov, A. A.; Heck, A. J. R., Ultralong transients enhance sensitivity and resolution in Orbitrap-based single-ion mass spectrometry. *Nat Methods* **2024,** *21* (4), 619-622.
3. Worner, T. P.; Aizikov, K.; Snijder, J.; Fort, K. L.; Makarov, A. A.; Heck, A. J. R., Frequency chasing of individual megadalton ions in an Orbitrap analyser improves precision of analysis in single-molecule mass spectrometry. *Nat Chem* **2022,** *14* (5), 515-522.
4. Wang, H. Z.; Song, M. Z., Ckmeans.1d.dp: Optimal-means Clustering in One Dimension by Dynamic Programming. *R J* **2011,** *3* (2), 29-33.
